# Immune Checkpoint Inhibitors for Pediatric Cancers: Is It Still a Stalemate?

**DOI:** 10.3390/ph17080991

**Published:** 2024-07-26

**Authors:** Tang-Her Jaing, Yi-Lun Wang, Chia-Chi Chiu

**Affiliations:** 1 Division of Hematology and Oncology, Department of Pediatrics, Chang Gung Memorial Hospital, 5 Fu-Shin Street, Kwei-Shan, Taoyuan 33315, Taiwan, China; g987669@gmail.com; 2 Division of Nursing, Chang Gung Memorial Hospital, 5 Fu-Shin Street, Kwei-Shan, Taoyuan 33315, Taiwan, China; chi0105@cgmh.org.tw

**Keywords:** immune checkpoint inhibitors, pediatric cancer, hypermutated, SMARCB1-deficient

## Abstract

The knowledge surrounding the application of immune checkpoint inhibitors (ICIs) in the treatment of pediatric cancers is continuously expanding and evolving. These therapies work by enhancing the body’s natural immune response against tumors, which may have been suppressed by certain pathways. The effectiveness of ICIs in treating adult cancers has been widely acknowledged. However, the results of early phase I/II clinical trials that exclusively targeted the use of ICIs for treating different pediatric cancers have been underwhelming. The response rates to ICIs have generally been modest, except for cases of pediatric classic Hodgkin lymphoma. There seems to be a notable disparity in the immunogenicity of childhood cancers compared to adult cancers, potentially accounting for this phenomenon. On average, childhood cancers tend to have significantly fewer neoantigens. In recent times, there has been a renewed sense of optimism regarding the potential benefits of ICI therapies for specific groups of children with cancer. In initial research, individuals diagnosed with pediatric hypermutated and SMARCB1-deficient cancers have shown remarkable positive outcomes when treated with ICI therapies. This is likely due to the underlying biological factors that promote the expression of neoantigens and inflammation within the tumor. Ongoing trials are diligently assessing the effectiveness of ICIs for pediatric cancer patients in these specific subsets. This review aimed to analyze the safety and effectiveness of ICIs in pediatric patients with different types of highly advanced malignancies.

## 1. Introduction

The main focus of cytotoxic chemotherapy and targeted therapies is to specifically attack cancer cells. However, immune checkpoint inhibitors (ICIs) function by enhancing the body’s immune response against tumors by interfering with co-inhibitory T-cell signaling. Resistance is a common occurrence in patients receiving both conventional cancer treatments and targeted therapies. Nevertheless, a considerable portion of patients who undergo ICI treatment exhibit enduring responses, suggesting the presence of a lasting immunologic memory.

ICIs have completely transformed the way adult cancers are treated [1]. Following its approval in 2011, the cytotoxic T-lymphocyte antigen-4 (CTLA-4) antibody has been utilized for the treatment of metastatic melanoma; eight ICIs targeting the PD-1 pathway have so far received approval. Currently, these ICIs are utilized in the treatment of 18 distinct forms of cancer [2]. Regrettably, the pediatric experience has been notable, as early studies have shown limited effectiveness of ICIs in children. This study emphasizes the distinction in immune response between pediatric and adult cancers, prompting a renewed emphasis on identifying particular subsets of pediatric tumors that may exhibit a greater level of responsiveness to ICI therapies [3]. We thoroughly explore the mechanisms of ICIs, analyze their utilization in both adult and pediatric cancers, and investigate two potential avenues for advancing the use of ICIs in pediatric patients: hypermutated cancers and SMARCB1-deficient tumors.

One example of an ICI is ipilimumab, a completely humanized monoclonal antibody. It focuses on CTLA-4, which is responsible for suppressing the immune response. In 2011, the US Food and Drug Administration (FDA) approved the utilization of ipilimumab in the treatment of advanced metastatic melanoma [4]. Treatment was continued until there was radiographic progression, unacceptable toxic effects, a decision made by the investigator, or the patient withdrew their consent [5,6,7,8].

## 2. Exploring the Biological Role of CTLA-4 and PD-1

The identification of CTLA-4 as part of the immunoglobulin gene superfamily in activated CD8 T cells marked a major advancement in the study of immune co-inhibitory molecules. In 1987, the cloning and identification of it was successfully achieved by Jean-François Brunet et al. [9]. In 1991, Jeffrey Ledbetter and Peter Linsley conducted a study that explored the interaction between CD28 and B7 in BMS. Their research indicated that CTLA-4 exhibits a higher binding preference for B7 in comparison to CD28 [10]. In the following year, the groups led by Jeffrey Bluestone and Peter Linsley separately conducted in vivo studies. The studies demonstrated that CTLA4-Ig, a soluble variant of CTLA-4, can extend the lifespan of islet grafts and inhibit the antibody response that is not dependent on T cells. One possible role for CTLA-4 is to regulate T-cell activity in a negative regulatory manner [11,12]. In 1994, Jeffrey Bluestone conducted research that highlighted the important role of CTLA-4 in suppressing the response of T cells [13]. It was demonstrated that the expression of CTLA-4 by activated T cells resulted in the direct binding to B7 on antigen-presenting cells, leading to a decrease in T-cell proliferation and function. James Allison conducted an independent study on the function of CTLA-4 and published his findings in 1995. He discovered that CD28 and CTLA-4 both interact with the ligand B7, but they have contrasting effects on T-cell functionality [14]. In 1995, two different research teams, the Tak Mak group and the Arlene Sharpe group, successfully created mice with a CTLA-4 gene knockout. The mice displayed a notable lymphoproliferative disorder which resulted in premature mortality, indicating that CTLA-4 plays a crucial role in the activation and proliferation of T cells [15,16].

The cloning and functional analysis of the PD-1 gene followed a similar procedure to that of CTLA-4. In 1992, Tasuku Honjo discovered the PD-1 gene by cloning it from a stimulated mouse T-cell hybridoma. This gene belongs to the immunoglobulin gene superfamily [17]. Later on, the Honjo group successfully created mice with a PD-1 gene knockout. However, in contrast to the mice lacking the CTLA-4 gene, the anticipated alterations did not occur rapidly. After a few months, as they aged, the C57BL/6 mice had glomerulonephritis and arthritis similar to lupus. [18]. When compared to T cells from wild-type mice, those obtained from animals lacking the PD-1 gene showed far more activated behavior. This finding supports the notion that PD-1 functions as a co-inhibitory molecule. In 2001, a study documented the presence of an autoimmune disease, specifically cardiomyopathy, in PD-1 gene knockout mice with BALB/c background [19]. Research findings indicate that PD-1 exerts a suppressive effect on the activation of T cells. It is worth mentioning that CTLA-4 and PD-1 may have varying functions, likely because of their unique mechanisms of action. We proceeded to search for a genuine PD-1 ligand that exhibits both physical interaction and inhibitory properties. The PD-L1 ligand was identified by the Lieping Chen group, while the PD-L2 ligand was identified by the Drew Pardoll group [20,21]. Extensive research conducted by Gordon Freeman and his team has revealed that the interaction between receptors and ligands, specifically PD-1/PD-L1 and PD-1/PD-L2, plays a significant role in the negative regulation of T-cell responses [22,23]. In 2004, two separate teams, the Lieping Chen group and the Arlene Sharpe group, successfully generated PD-L1 gene knockout mice [24,25,26]. Mice lacking the PD-L2 gene were created in 2006 by research groups led by Chen Dong and Arlene Sharpe [27,28,29]. It was shown that both ligands may bind directly to PD-1 and improve tolerance of T-cell activation.

**Figure 1:**
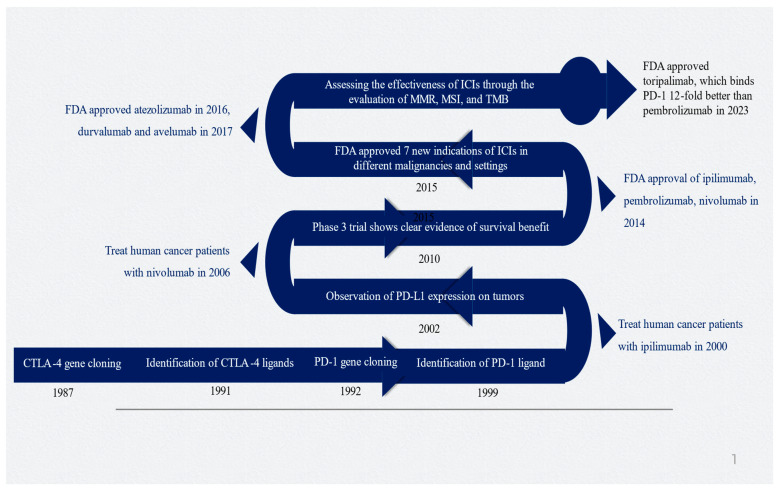
Timeline of critical milestones for developing immune checkpoint inhibitors. Abbreviations: CTLA-4, cytotoxic T-lymphocyte-associated protein 4; ICI, immune checkpoint inhibitor; MMR, mismatch repair; MSI, microsatellite instability; PD-1, programmed death-1; PD-L1, programmed death-ligand 1; TMD, tumor mutational burden. Figure generated by authors based on the existing literature [9,10,30,31,32,33].

James Allison and Tasuku Honjo made significant contributions to cancer immunotherapy with their research on CTLA-4 and PD-1, which led to them being awarded the esteemed 2018 Nobel Prize in Physiology and Medicine [30]. Figure 1 depicts the timeline and important milestones in the development of ICIs.

## 3. Mechanism of Action of Immune Checkpoint Inhibitors

ICIs function by augmenting the body’s inherent T-cell responses against tumors, which might have been compromised by inhibitory pathways. T-cell responses primarily target neoantigens, which are proteins formed due to genetic mutations in cancer cells. This phenomenon occurs as a result of their inherent capacity to elicit an immune response [3]. Neoantigens, like viral antigens, are not found in the normal human proteome, which causes T cells to recognize them as foreign. Upon encountering tumor antigens in the draining lymph nodes, the T cells that target the tumor undergo activation. Migrating to the tumor microenvironment, clonal proliferation, and enhanced effector activity (including cytokine production and cytolytic capability) are all outcomes of the process. When T cells arrive at the tumor site, they come across tumor antigens, which then prompt their activation and subsequent removal of tumor cells. However, once a patient shows signs of disease, their T-cell responses have already been greatly suppressed by different inhibitory pathways. These pathways involve the activation of immune checkpoints, which are types of inhibitory receptors. Immune checkpoints play a crucial role in regulating the activity of tumor-specific T cells, preventing them from effectively eliminating cancer cells. The ICIs work by disrupting the signaling of inhibitory immune checkpoints, which in turn allows tumor-specific T cells to be activated and promotes an immune response against the tumor.

**Figure 2:**
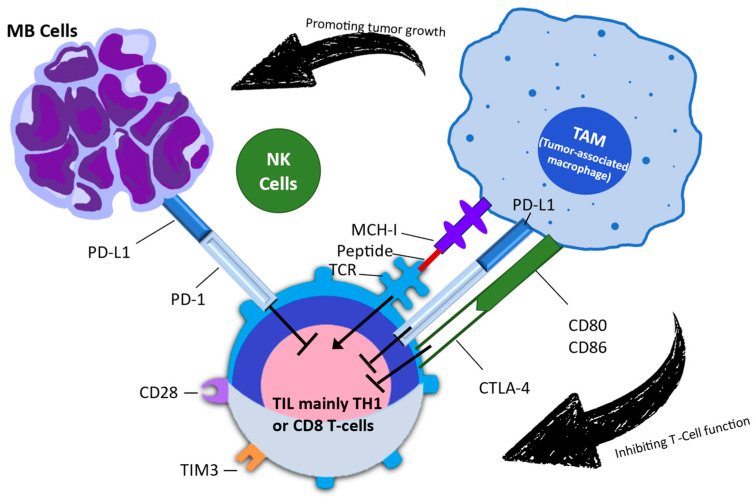
This figure illustrates the intricate signaling interactions between tumor cells, tumor-associated macrophages, and tumor-infiltrating lymphocytes within the medulloblastoma microenvironment. This figure is based on the research conducted by Kurdi et al. (2023) [43]. MB: medulloblastoma; NK: natural killer; TAM: tumor-associated macrophage; TIL: tumor-infiltrating lymphocyte; TIM-3: T-cell immunoglobulin and mucin domain 3.

Due to the significant harm caused by conventional therapies, particularly in the developing brains of children, there is a pressing demand for improved treatment strategies. Immunotherapy, which has garnered increasing attention over time, offers a potential solution. Information is scarce regarding the potential application of immunotherapy in brain tumors. While children have generally tolerated ICIs well, their response to these treatments has been extremely limited or nonexistent. Figure 2 depicts the signaling interaction between tumor cells, tumor-associated macrophages, and tumor-infiltrating lymphocytes in the medulloblastoma microenvironment [34]. The initial immune checkpoints that were identified were CTLA-4 and PD-1. Only CTLA-4 and PD-1 have been successfully targeted in clinical settings, despite the discovery and ongoing investigation of several more checkpoints [35]. CTLA-4 is an inhibitory molecule that becomes more active on T cells following the first encounter with an antigen, helping to restrict T-cell activation at this early stage. To achieve successful T-cell activation during priming, two signals are required from antigen-presenting cells. The first signal involves T-cell receptor signaling, which occurs when the T-cell interacts with the antigen in the major histocompatibility complex. The second signal pertains to costimulation via CD28, which occurs when the T-cell engages with B7-1 (CD80) or B7-2 (CD86). Upon receiving these two signals, activated T cells increase the expression of CTLA-4 as a means to regulate excessive activation [36].

CTLA-4 is structurally similar to CD28, but it binds B7-1 and B7-2 ligands more strongly [37]. CTLA-4 expression efficiently outperforms CD28 binding to its ligands and controls the costimulatory signal. This passage discusses how monoclonal antibodies can inhibit the interaction between CTLA-4 and CD28 ligands, resulting in increased effectiveness and duration of T-cell activation. The tumor microenvironment displays increased clonal diversity of tumor-specific T cells, enhanced T-cell migration to tumors, and a higher ratio of effector-to-regulatory T cells. CTLA-4 is an immunological regulator. It interacts with the B7 ligand on antigen-presenting cells, which plays a role in T-cell suppression. The evasion of the immune response against tumor cells can be achieved by inhibiting the activity of cytotoxic T-cells through the CTLA-4/B7 checkpoint. Research suggests that CTLA-4 plays a role in suppressing T-cell activation by affecting T-cell receptor (TCR) signaling, altering CD28 localization, and sequestering B7 ligands from antigen-presenting cells (APCs). CTLA-4 inhibitors function by blocking T-cell inhibitory signals, resulting in heightened T-cell activity and proliferation. This ultimately strengthens the immune response against cancer cells [38].

Activated T cells, B cells, and monocytes are among the immune cells that contain PD-1. It has a significant impact on suppressing the immune system’s response. PD-L1 and PD-L2 ligands are detectable on both antigen-presenting cells (APCs) and tumor cells. The interaction between PD-L1 and PD-1 on T cells leads to the suppression of their function, enabling tumor cells to evade detection by the immune system. The inhibitors impede the interaction, thus promoting the activation of cytotoxic T-cells against the tumor cells. Tumors with heightened PD-L1 expression can effectively evade the immune system and hinder the body’s ability to respond to T cells that combat cancer. One way in which PD-1/PD-L1 inhibitors work is by preventing the immune system from tolerating tumor cells [39].

PD-1 hinders T-cell activity in peripheral tissues following prolonged exposure to antigens, in contrast to CTLA-4 which primarily suppresses T cells during the initial activation process. Prolonged exposure to antigens in the tumor microenvironment leads to T-cell fatigue. This process results in the reprogramming of T cells and a gradual decrease in their effector functioning as time goes on. PD-1 expression is responsible for the development of T-cell exhaustion. This protein helps bring inhibitory phosphatases to the immune synapse by binding to its ligands PD-L1 and PD-L2. This changes the communication between T-cell receptors. The endothelium is one of several tissues that often contain PD-1 ligands. However, in certain instances, cancers can also display elevated levels of PD-L1 [40]. The recruitment of suppressive myeloid cells expressing PD-L1 to the tumor microenvironment can occur, effectively exploiting a pathway that typically aids in preventing immunopathology in healthy tissue. This passage provides a clear and scholarly explanation of how monoclonal antibodies can improve T-cell function and empower them to fight against tumors by inhibiting the PD-1/PD-L1 interaction. It is important to highlight that PD-1 functions differently from CTLA-4, which offers a scientific justification for using ICIs that target both pathways concurrently. Significant progress is being made in the field of brain cancer treatment, both in preclinical and clinical environments. New therapies targeting emerging factors like T-cell immunoglobulin, mucin domain 3 (TIM-3), and indoleamine 2,3-dioxygenase-1 (IDO1) are showing promising results. In addition, there is promising potential in agonistic therapies that focus on the positive stimulatory immune checkpoint CD40 [41,42]. 

### 3.1. Experience with Immune Checkpoint Inhibitors in Pediatric Cancers

The most extensive research with immune checkpoint inhibitors in pediatric cancers has focused on the PD-1 axis. Childhood cancers are often classified as “cold” tumors because of their low tumor mutational burden (TMB), PD-1 expression, and T-cell infiltrates. Four studies conducted between 2020 and 2022 present the most data on the effectiveness of different immunotherapy drugs as monotherapies for recurrent and refractory pediatric tumors [44]. All four studies found that the ICIs were well tolerated by children when administered at weight-based dosing that aligned with the approved adult doses. The dosages used were 3 mg/kg of nivolumab administered every 2 weeks, 2 mg/kg of pembrolizumab given every 3 weeks, 15 mg/kg of atezolizumab administered every 3 weeks, and 20 mg/kg of avelumab given every 2 weeks [45,46]. The adverse events observed in pediatric patients were similar to those seen in adults, except for a higher incidence of cytopenia, including grades 3–4. This could be attributed to the higher level of pretreatment in pediatric patients compared to adults. Furthermore, the most common adverse events observed were constitutional symptoms such as fatigue and fever, in grades 1 and 2. The most prevalent adverse event connected to the immune system was shown to be liver toxicity, most especially increased transaminases grades 1 and 2. In addition, the following immune-related side effects have been reported on rare occasions: colitis, pleural and pericardial effusions, thyroiditis, pancreatitis/elevated lipase, and thyroiditis. Out of the 350 pediatric patients examined in these studies, it is important to highlight that two individuals experienced notable toxicities that may be linked to the treatment.

Immunotherapy drugs targeting PD-1/PD-L1 have demonstrated notable benefits in treating pediatric classic Hodgkin lymphoma, with the disease’s biology playing a crucial role in determining treatment response. Reed–Sternberg cells often display pathological instances of chromosome 9p24.1 mutations, resulting in high PD-L1 and PD-L2 levels [47]. Hodgkin lymphoma’s genetic susceptibility makes it highly responsive to checkpoint blockade, enhancing its potential for treatment. In pediatric patients with relapsed Hodgkin lymphoma, PD-1/PD-L1-targeting ICIs demonstrated an objective response rate of 30% to 60%, similar to that observed in adults. The current FDA approval for pembrolizumab includes this indication. 

Despite the various obstacles, there is cause for optimism regarding the potential benefits that children with cancer may experience as a result of advancements in oncology, particularly in the areas of immune amplifiers and ICIs. At the outset, it is observed that pediatric tumors have relatively low mutation burdens upon initial diagnosis. However, it has been noted that the frequency of mutations tends to rise with the administration of chemoradiotherapy [48,49]. Neoantigens can arise from either direct production or mutations in mismatch repair genes. Exploring the combination of checkpoint inhibition and agents that promote non-synonymous somatic mutations (NSSMs) shows great potential in the study of adult cancers. The potential of these NSSMs is significant, as they can facilitate the development of focused therapeutic approaches. Through the identification of precise mutations in cancer cells, researchers can develop drugs that specifically target these mutations. This advancement enables the creation of treatment options that are both more effective and tailored to individual patients [50,51,52,53]. This strategy holds potential for children as well, with a planned study in neuroblastoma (Clinicaltrials.Gov: NCT02914405). Research has shown that radiation can affect the growth of cancers, leading to the emergence of new targets for immune cells involved in tumor suppression [54,55]. In addition, radiation has the potential to improve immune activation and enhance the response to checkpoint blockade. This can be achieved through various mechanisms, including the augmentation of neoantigens. In some intriguing situations, a metastatic location outside of the radiation field was able to regress due to an abscopal effect caused by a combination of targeted radiation and checkpoint blockage [56]. In their study, Leruste and colleagues made an interesting discovery regarding rhabdoid tumors. It was discovered that these tumors have a notable abundance of T cells, including worn-out effector and expanded memory CD8 populations [57]. In addition, there is a high presence of immunosuppressive myeloid-derived cells in these tumors [58].

There is limited experience in targeting CTLA-4 with ipilimumab in pediatrics. Ipilimumab was considered safe, but it appeared to result in more immune-related adverse events compared to PD-1/PD-L1 ICIs, especially at higher doses, similar to those in adults [3]. The FDA has authorized the use of ipilimumab to treat metastatic or unresectable melanoma in children and adolescents over the age of 12, based on these findings and by extending adult data to pediatric patients with comparable tumor characteristics [59]. On the other hand, nivolumab has been approved for a wider range of cancers compared to ipilimumab [60].

**Figure 3:**
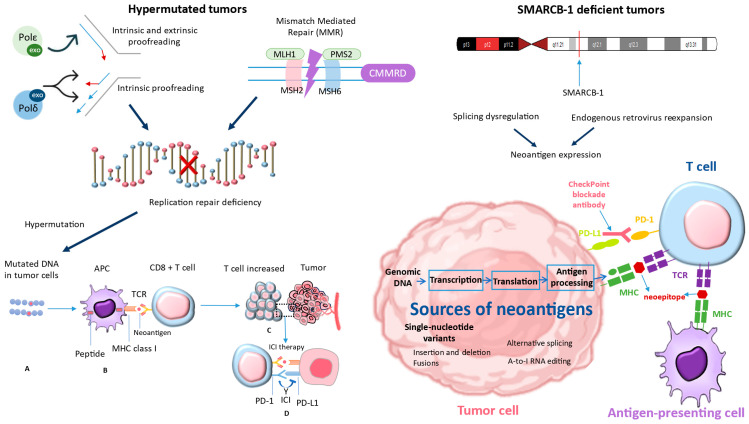
The studies conducted by Davis et al. (2021) [64] were used to derive the bottom-right corner of Figure 3, while the left part of Figure 3 was derived from Long et al. (2022) [3]. These studies demonstrated that tumors with deficiencies in MMRD and SMARCB-1 have reignited interest in the potential use of ICIs in these patients. MMRD: mismatch repair deficiency; SMARCB-1: SWI/SNF-Related, Matrix-Associated, Actin-Dependent Regulator Of Chromatin, Subfamily B, Member 1.

Recent studies have shifted their focus to combination ICIs, highlighting the additional benefits observed in adult patients and the biological rationale behind the synergistic effects of CTLA-4 and PD-1 blockade [61]. Regrettably, up to now, the ipilimumab/nivolumab combination does not appear to offer enhanced effectiveness against pediatric solid tumors [62]. When it comes to children, why do ICIs not work as well? Although PD-L1 expression is still rare in pediatric malignancies, there is minimal indication that it is a crucial factor. The apparent lack of effectiveness indicates a fundamental distinction in the immunobiology of cancers in children and adults. Childhood cancers are often categorized as “cold tumors”, known for having low mutational burdens and fewer infiltrating T cells [63]. Ongoing research into the immunobiology of pediatric cancers aims to improve the utilization of immunotherapies for these patients. While ICIs may not be beneficial for most pediatric cancer patients, recent data have identified specific patient groups that may respond well to ICIs. Patients with distinct tumor biology often exhibit increased neoantigen burdens or greater numbers of infiltrating immune cells. Interest has been sparked in tumors with MMRD and SMARCB-1 deficiencies, reigniting enthusiasm for the potential use of ICIs in these patients (Figure 3). Patients with hypermutated tumors, including those with alterations in SMARCB1, may have a more favorable immune response to certain treatments like ICIs if they have neoantigen-reactive T cells [64]. Tumors that have undergone hypermutation may have a higher chance of responding to these therapies [3]. This is because they have a wide range of neoantigens that can be targeted by the activated T cells.

The mutational burden of a tumor is an inherent characteristic that is associated with the immune response against the tumor and its response to ICI. This correlation is likely due to the increased formation of neoantigens resulting from a higher number of non-synonymous single-nucleotide variants [65]. Microsatellite instability due to a lack of DNA mismatch repair and an improved response to PD-1 inhibition have been linked in studies [66]. 

ICIs may not be very effective in the general pediatric cancer population, except for Hodgkin lymphoma. The overexpression of PD-L1 due to the amplification of a specific region on chromosome 9p24.1, which includes PD-L1, PD-L2, and JAK2, has been observed in Hodgkin lymphoma. This overexpression is believed to be responsible for the significant clinical response to PD-1 blockade [67]. However, there is a possibility of higher response rates in specific subgroups. This subgroup includes tumors that have a mismatch repair deficiency caused by mutations or epigenetic silencing of certain genes, as well as a polymerase-proofreading deficiency caused by mutations in other genes. The identified deficiencies result in hypermutation and the subsequent generation of neoantigens. These neoantigens have the potential to elicit a response from T cells. One possible subgroup comprises tumors that lack the SMARCB1 gene. Various mechanisms have been proposed, such as dysregulation of splicing, re-expression of endogenous retroviruses leading to neoantigen expression, and enhanced infiltration of tumor immune cells. These processes may be interconnected or influenced by other yet unknown factors [3]. Immunotherapy medications, such as anti-PD-1, enhance the body’s defenses against malignancies by blocking the PD-1–ligand interaction. Consequently, the tumor’s T cells become more activated.

### 3.2. Exploring Immune Checkpoint Inhibition in Hypermutated Cancers

Pediatric cancers generally exhibit a lower mutational burden in comparison to adult cancers, particularly carcinomas. This difference is likely due to the distinct processes of carcinogenesis [68]. Certain types of adult cancers, such as melanoma, non-small cell lung cancer, and bladder cancer, are particularly responsive to ICI therapies. These cancers typically have a significant number of genetic mutations. Multiple studies have found a correlation between higher TMB and positive outcomes in adult patients with melanoma, non-small cell lung cancer, and colorectal cancer. These outcomes include sustained clinical improvement, favorable radiologic response, and extended periods without disease progression [69,70]. On the other hand, the relatively small number of mutations found in pediatric cancers indicates a reduced presence of neoantigens. As a result, there is a higher chance that the immune system will not identify the tumor as foreign. The relatively low TMB observed in most pediatric cancers may explain the limited efficacy of ICIs in the overall pediatric cancer population [71].

It is crucial to note that the majority of pediatric cancers exhibit a low TMB. However, there is a small subset that shows a significantly higher mutational burden. Among the nearly 3000 pediatric cancers that were examined, a small fraction (5%) displayed a significant TMB (tumor mutational burden) exceeding 10 mutations per megabase [3]. A minuscule fraction (1%) fell under the category of ultra-hypermutated, displaying a TMB surpassing 100 mutations per megabase. Replication repair deficiency can lead to high mutational burdens. This occurs when crucial genes responsible for maintaining the integrity of DNA replication undergo mutations [72]. Replication repair deficiency arises from deficiencies in two key mechanisms: DNA MMR and DNA polymerase proofreading [73]. These mechanisms play a crucial role in ensuring precise replication, either individually or in conjunction. Hypermutated cancers often exhibit a deficiency in DNA MMR, a crucial surveillance system that corrects errors in base-pairing during the process of DNA replication. MMRD is the result of genetic mutations in the MMR genes, namely MLH1, MSH2, MSH6, and PMS2 [74]. In addition, it is worth noting that ultra-hypermutated tumors frequently exhibit a combination of MMRD and polymerase-proofreading deficiency. It is usual to perform this by introducing extra mutations into polymerases ε or δ (POLE or POLD1) [75]. Without proper proofreading and surveillance systems in place, there is a rapid accumulation of additional mutations, resulting in a substantial increase in TMB. Ultimately, this leads to the emergence of numerous new neoantigens capable of eliciting an immune response.

### 3.3. Normal and Malignant Human Cells Have Different Microsatellite Instability Signals from DNA Polymerase and Mismatch Repair

Both somatic and germline mutations can give rise to tumor MMRD and polymerase-proofreading deficiency. Certain types of cancers that affect the gastrointestinal and genitourinary systems can be attributed to specific genetic mutations. These mutations can occur in genes like POLE and POLD1, as well as in MMR genes, which are associated with Lynch syndrome [76]. Moreover, the lack of MMR function in the germline results in a notable cancer predisposition syndrome known as constitutional MMRD [77]. This syndrome is characterized by the early onset of various types of cancers, including high-grade gliomas, gastrointestinal malignancies, and hematologic malignancies. Cancers often develop in individuals with constitutional MMRD as a result of early somatic-driver mutations in POLE or POLD1 [78]. Hence, mutations can accumulate quickly, with some cases even reaching up to 600 mutations per cell division. Managing patients with these conditions can pose significant challenges due to the aggressive nature of their malignancies, limited response to chemotherapy, and the increased likelihood of concurrent cancer diagnoses. Immunohistochemistry plays a crucial role in the examination of suspected cases. Patients with constitutional MMRD demonstrate a deficiency in MMR protein expression in both tumor and normal tissues. Nevertheless, it is worth noting that individuals with Lynch syndrome will only exhibit a deficiency in MMR expression within tumor cells [79].

An effective way to assess MMRD is by analyzing its influence on MSI, rather than focusing on the broader measurement of TMB [80]. MSI assessment has predominantly been utilized in the examination of adult cancers. Typical panels utilized for MSI testing focus on a restricted set of microsatellites, which are short tandem repetitive DNA sequences. If multiple loci exhibit changes, the tumor is categorized as MSI-high. If a single sequence undergoes mutation, it is categorized as MSI-low [81]. Based on the current circumstances, the tumor is categorized as microsatellite-stable. Nevertheless, the reliability of panel testing might be compromised when conducted on pediatric patients with constitutional MMRD. Panel testing was employed to assess MSI and revealed that a minority of constitutional MMRD cancers displayed MSI-high features. Further investigation of MSI in childhood constitutional MMRD cancers has uncovered the occurrence of microsatellite insertions or deletions throughout the entire genome, rather than being limited to the typical five loci. It has been established that these types of cancers exhibit MSI accumulation, albeit at varying locations. It is crucial to acknowledge that variations in loci can result in a false-negative outcome on panel testing. Caution should be exercised when using standard MSI methodologies to assess the occurrence of MMRD in pediatric cancers.

### 3.4. Immune Checkpoint Inhibition for Hypermutant Tumors

A report published in 2016 examined the use of ICIs in the treatment of pediatric patients with refractory hypermutated cancers [82]. The case study focused on two brothers who were diagnosed with recurrent multifocal glioblastoma with constitutional MMRD. It also mentioned that they had positive responses to nivolumab. Many children worldwide with constitutional MMRD have received ICIs as a treatment for recurring illnesses when other options are not feasible. The International Replication Repair Deficiency Consortium recently collected data from these patients, which have confirmed significant response rates. The study included a total of 38 patients who were given either nivolumab or pembrolizumab as treatment [83]. Of the 45 cancers examined, 25 tumors, or 55.5% of the cases, showed a positive response in the form of objective responses or stable disease. In patients who underwent a central radiology review, the objective response rate was 42%. Out of this, 17% achieved a complete response, while 25% achieved a partial response. Patients with progressive or recurrent malignancies of the central nervous system had a somewhat worse 3-year survival rate of 39.3%, compared to the total group survival rate of 41.4%. This represents a substantial improvement compared to previous projections. An investigation was carried out to explore the relationship between genomic biomarkers and outcomes following ICI therapy [76]. The study enrolled patients who had germline replication repair deficiency, such as Lynch syndrome, constitutional MMRD, or POLE mutation. These patients also had confirmed tumor MMRD and elevated TMB. The study uncovered a connection between these biomarkers and the effectiveness of treatments. The study revealed a connection between a higher TMB (based on single-nucleotide variants) and enhanced tumor response and overall survival. The study found that the presence of both tumor MMRD and polymerase-proofreading deficiency had a significant impact on the outcomes, as opposed to MMRD alone. Furthermore, it is important to highlight that a strong correlation was found between a higher occurrence of microsatellite insertions or deletions and a favorable response, as well as enhanced overall survival. It is worth noting that this correlation was only observed in tumors with MMRD alone, which also showed a slightly lower TMB. In contrast, there was no observed correlation in tumors that exhibited both MMRD and polymerase-proofreading deficiency. A prediction model study for both replication repair-deficient cancer types showed a good prognosis associated with total microsatellite insertions or deletions and a high number of single-nucleotide variations. 

The FDA has approved the use of nivolumab and the combination of nivolumab/ipilimumab in the treatment of recurrent metastatic colorectal cancer. This approval is specifically for patients aged 12 and above who have been designated as having MMRD or MSI-high. For the treatment of recurrent advanced solid tumors with MSI-high designation or MMRD, pembrolizumab received expedited approval by the FDA in 2017, regardless of histology. This approval was extended in June 2020 to include advanced solid cancers with a high TMB of above 10 mutations per megabase. This milestone marked the initial FDA approval predicated on TMB. The approvals were given after the data from the adult phase II KEYNOTE-158 study were released. The study revealed a 34.3% objective response rate in non-colorectal solid tumors classified as MSI-high or with MMRD. Furthermore, the rate of response stood at 29% among patients with hypermutated tumors, characterized by a TMB exceeding 10 mutations per megabase. 

In general, there are promising data that support the utilization of ICIs in children who have hypermutated cancers associated with MMRD [84]. The response rates in patients with ultra-hypermutated cancers are particularly noteworthy. These cancers are often linked to constitutional MMRD and the subsequent acquisition of a somatic POLE/POLD1 mutation, which results in the rapid accumulation of mutations. It remains uncertain how tumors with a lower number of mutations (10–100 or even 5–10 mutations per megabase) respond, and whether therapy-induced hypermutation can predict response in the same manner as hypermutation caused by MMRD. It should be noted that in the field of adult malignancies, there seems to be a correlation between the precise underlying cause of high TMB, such as MMRD or polymerase-proofreading deficiency mutation, and better treatment results after ICI therapy. Nevertheless, the increased TMB does not appear to have a direct impact on the enhanced outcome [81]. Additional analysis reveals that the correlation between higher TMB and the efficacy of ICIs is absent in cancer types where TMB does not correspond to neoantigen load or CD8 T-cell infiltration. Hence, while TMB can provide valuable insights into patient eligibility, it is crucial to take into account additional factors, particularly those related to the tumor immune environment, when evaluating the possible effectiveness of ICI therapy. Currently, it is not advisable to suggest routine MSI or TMB testing for all pediatric patients with cancer. If a patient is going through panel, whole exome, or genome sequencing and has a recurrence, it is essential to assess TMB. Patients with constitutional MMRD-associated malignancies or Lynch syndrome must also have TMB testing, even though these cases are very uncommon.

## 4. How the Body Develops Intolerance to ICI Treatment

Our understanding of the mechanisms behind innate and acquired resistance to ICI therapy is still incomplete. One contributing factor to this is the lack of comprehensive understanding regarding the various clinical, molecular, and immunologic factors associated with the clinical response and long-term advantages of ICI therapy. It is commonly recognized that a successful immune response against tumors after PD-1/PD-L1 blockade requires the reactivation and clonal proliferation of T cells with previous antigen exposure in the tumor microenvironment (TME) [85]. To ensure durable immunologic memory and sustain efficient disease control, a particular subset of effector T cells must undergo differentiation into effector memory T cells. The process is facilitated by the help of CD4+ helper T cells and dendritic cells. The organisms remain viable throughout their entire lifespan and demonstrate a reaction when exposed to antigens [86]. Tumors employ multiple mechanisms within their structure to evade the immune system. The mechanisms encompass genetic and epigenetic alterations that impact the creation, presentation, and processing of neoantigens. In addition, changes in cellular signaling pathways can interfere with the function of cytotoxic T cells. Non-cancerous stromal or immune cells, as well as other systemic effects, may collaborate with cancer cells to enhance tumor development and resistance to ICI; this process is known as creating tumor-extrinsic pathways. 

**Figure 4:**
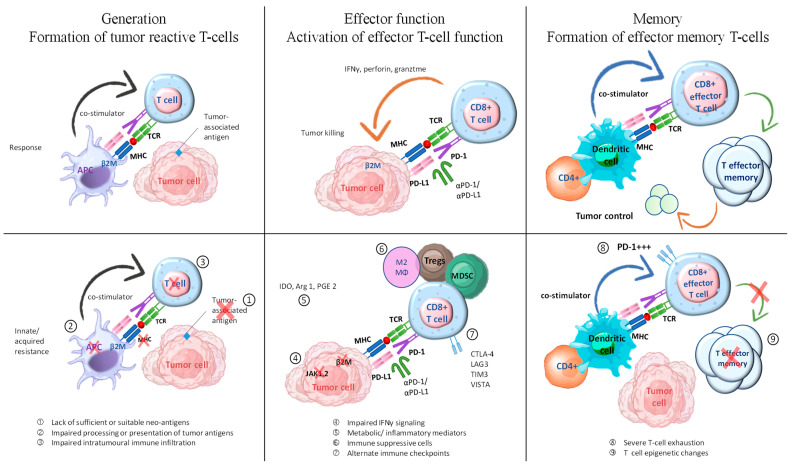
Exploring combination therapies to address resistance in ICI therapy. The process of generating an effective tumor-directed T-cell response involves several steps. These include the formation of tumor-specific T cells, the activation of effector T-cell function, and the development of effector memory T cells. This figure is derived from the study of Fujiwara et al. (2020) [33].

Failure of ICI therapy can occur due to deficiencies in any of the mentioned steps, which can be categorized into three distinct groups: (1) insufficient generation of anti-tumor T cells, (2) inadequate functioning of tumor-specific T cells, and (3) issues with the formation of T-cell memory are potential factors to consider (Figure 4) [33]. Tumor-reactive T-cell formation can be impeded by multiple factors, including inadequate or inappropriate neoantigens, compromised neoantigen processing, and impaired neoantigen presentation [87]. Various methods can be employed to promote immunogenic cell death, such as chemotherapy and radiation. Additionally, activating innate immune responses and enhancing dendritic cell function can help increase antigen presentation. These approaches have the potential to facilitate the development or display of suitable antigens in tumors that lack immune cells and have a non-inflamed tumor microenvironment. In addition, inhibiting immunosuppressive factors could enhance dendritic cell migration, maturation, and function. This could potentially improve T-cell priming and collaboration with ICIs. 

Checkpoints or co-inhibitory receptors on the immune system, cytokines that suppress the immune system, metabolites that inhibit the immunological system, and cells that suppress the immune system are all components of immune evasion. Numerous combination therapies are currently under development to tackle innate resistance by focusing on potential immune evasion mechanisms within the TME. 

## 5. Adverse Events of Immune Checkpoint Inhibitors

ICIs can lead to immune system overstimulation, which in turn raises the potential for immune-mediated toxicity. Although the exact physiopathology is still unclear, there exist several hypotheses regarding the underlying mechanism responsible for the development of immune-related adverse events (irAEs) [88]. ICIs are widely regarded as a safer alternative to chemotherapy due to their lower incidence of severe side effects. However, it is important to note that ICIs still carry some risks, including the possibility of serious immune-related adverse events. Studies estimate that around 2% of treatment-related deaths can be attributed to ICIs [89]. The pathophysiology of irAEs may differ based on the specific organ involved. Neurological adverse events can be attributed to the release of onconeural peptides that occur when cancer cells die. They initiate the production of onconeural autoantibodies, which can potentially result in harm to the organs [90].

Patients with a variety of advanced malignancies are now being treated with ICIs, which has led to remarkable improvements in their overall health. Nevertheless, when ICIs stimulate the immune system, they also inadvertently disrupt the immune balance in non-tumor tissues, causing significant immune and inflammatory responses. These encompass clinical immune-related adverse events. ICIs are effective in treating tumors, but it is worth noting that a significant percentage of patients, ranging from 30% to 60%, may experience irAEs. Immune checkpoint inhibitors have shown effectiveness in treating different types of cancer, but they can cause specific adverse events related to the immune system in different organs [91]. Immune checkpoints are essential for regulating the T-cell immune response by functioning as negative regulators. The immune system relies on these chemicals to keep tolerance levels high and stop autoimmunity in its tracks. In contrast to the cytotoxic effects of traditional chemotherapy, which occur in cycles and primarily impact rapidly dividing cells, irAEs present as sporadic and unpredictable manifestations that often target specific organs. The skin, colon, liver, endocrine glands, and lungs are frequently involved in this condition [92]. Typically, reactions of grade 3 or higher are deemed sufficient to warrant the permanent discontinuation of ICIs [93].

### 5.1. Pulmonary Toxicity of Immune Checkpoint Immunotherapy

Tumors can evade the immune system through different mechanisms. These include modifying or eliminating antigens, increasing the expression of immune checkpoint molecules, and manipulating cytokines and oncogenic signaling to establish an immune-suppressive environment within the tumor. There have been associations found between ICI therapy and various lung complications. Checkpoint inhibitor-related pneumonitis (CIP) is one of many possible side effects, along with sarcoid-like granulomatosis, pleural effusion, and worsening of obstructive lung disease. The development of CIP is characterized by increased immune activation mediated by T cells, imbalances in cytokine levels, the upregulation of autoantibodies, and genetic predispositions. These factors collectively contribute to inflammation in the lungs [94]. However, diagnosing CIP solely based on radiographic features is not possible. Several alterations have been detected by CT scans; as many as half of the cases documented in the literature show either ground-glass opacities or consolidative lesions. Research has explored genetic variations as a potential contributor to the occurrence of irAEs, which are frequently associated with autoimmune conditions.

### 5.2. Cellular Autoimmunity/Higher T-Cell Activity

**Figure 5:**
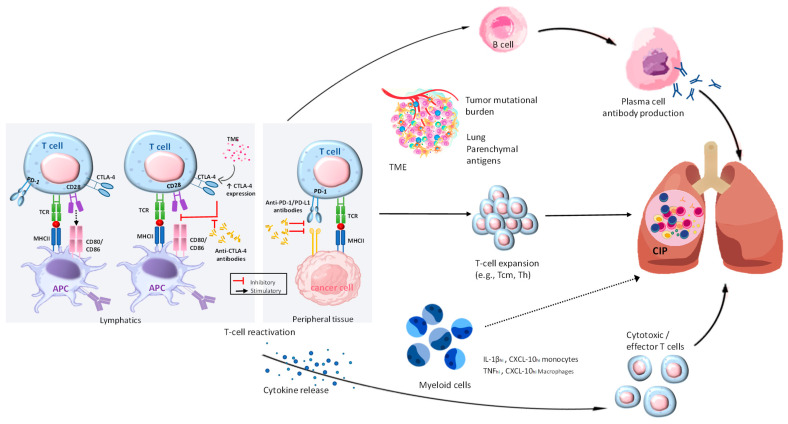
Exploring the pathophysiological mechanisms in CIP. By utilizing ICI, T cells can overcome the immunosuppression caused by cancer. The activation of different pathways leads to the proliferation of B and plasma cells, which in turn produce autoimmune antibodies like anti-CD74. In addition, they trigger the release of inflammatory cytokines such as IL-1β, TNF-α, and CXCL-10, which can affect various cell types. In addition, T cells, such as Tcm, Th, and clonal T cells, undergo expansion in response to various factors within the tumor microenvironment, the tumor’s mutational burden, and self-antigens located in the lung tissue. The combination of these various pathways can cause inflammation in the lungs, leading to CIP. The role of myeloid cells in CIP is clear, although the exact mechanisms are still not fully understood. Their role in T-cell activation and expansion can be influenced by the T-cell and cytokine environment, potentially leading to pulmonary injury. The presence of clear lines in CIP signifies the existence of established mechanisms, whereas the presence of dotted lines suggests potential mechanisms. This figure is derived from the study of Ghanbar et al. (2024) [94].

There is a growing body of evidence suggesting that the upregulation of T cells may be involved in the development of CIP, as illustrated in Figure 5. A thorough assessment of clinical symptoms and radiological findings is the main basis for the diagnosis of CIP. There are a wide variety of radiographic patterns that may be seen in chronic interstitial pneumonia (CIP), including cryptogenic organizing pneumonia (COP), hypersensitivity pneumonitis (HP), non-specific interstitial pneumonia, and acute interstitial pneumonia [95]. Treatment for CIP that exceeds grade 2 typically involves the use of high-dose corticosteroids. Recurrent CIP can occur following steroid treatment, regardless of the status of ICI administration.

### 5.3. Genetic Predisposition

Genetic variations have been extensively studied as a possible factor in the development of irAEs, which are frequently linked to autoimmunity. Multiple pathways may be involved in the complex interaction of various irAEs, as indicated by the association of several genes with single-nucleotide polymorphisms [96,97,98]. The variations in human leukocyte antigens hold immense importance due to their critical involvement in the interaction of immune cells.

## 6. Discussion

Autoimmune events, also known as irAEs, are commonly seen as complications of ICIs. Immune-related adverse events (IrAEs) arise from the activation of effector T cells and the suppression of the Treg function, leading to an inflammatory condition. Auto-reactivity in healthy organ tissue occurs as a result [99,100,101]. Checkpoint inhibitors are responsible for the majority of immune-related adverse events (irAEs) in cancer treatment [102].

A study revealed that a notable proportion of individuals who are prescribed ICIs experience adverse events. Furthermore, a notable proportion of individuals, ranging from 17 to 59%, experience severe grade 3 or 4 side effects, as described in the Common Terminology Criteria for Adverse Events (CTCAE). In this study, it was found that around 17% of patients who were given anti-PD-1 monotherapy experienced notable side effects. On the other hand, more than 59% of individuals who underwent ipilimumab and nivolumab therapy encountered grade 3 or 4 toxicities [103].

Monitoring guidelines are necessary because of the notable prevalence of immune side effects. In 2017, the Toxicity Management Working Group was established by the Society for Immunotherapy of Cancer (SITC). This group later published guidelines for monitoring adverse immune events. The group recommends conducting a variety of medical tests before treatment. These tests include blood count, metabolic panel, thyroid studies, cortisol and adreno-corticotropic hormone levels, hemoglobin A1c, electrocardiogram, and various other tests [104]. It is important to establish a foundation by repeating these steps before moving on to future cycles. The assessment of potential adverse events differs depending on the specific organ system involved. Colitis is frequently observed in individuals with cancer and can arise from either infectious or autoimmune causes when undergoing checkpoint inhibitor treatment. Crucial to a thorough evaluation is the use of stool samples for culture, parasites, C. difficile antigens, CMV polymerase chain reaction, inflammatory markers, CT scans, and colonoscopy, if necessary. Pneumonitis is a less common but important adverse event that is associated with higher mortality rates. It is recommended to include chest CT, pulmonary function testing, and a six-minute walking test in the diagnostic evaluation. The development of arthropathy in patients may warrant a comprehensive evaluation to investigate potential rheumatologic causes. Some of the tests that may be included in this examination are MRI, erythrocyte sedimentation rate, C-reactive protein, cyclic citrullinated peptide antibody, rheumatoid factor, and anti-nuclear antibody. Although uncommon, therapy might cause neurologic side effects including neuropathy, transverse myelitis, and aseptic meningitis. Brain MRI and lumbar puncture should be considered as part of the work-up [105].

The management of irAEs involves the use of corticosteroids to suppress the immune response. Recommended for more severe events, high-dose oral corticosteroids (0.5–2 mg/kg) have shown effectiveness as a treatment [99]. If steroids and withholding the checkpoint inhibitor do not yield desired results, more potent immunosuppressants may be considered. For instance, significant progress has been made in the management of irAEs through the utilization of TNF-alpha and calcineurin inhibitors [106,107,108].

Restarting ICIs in patients who have experienced an irAE carries both advantages and potential drawbacks. According to a retrospective study, a significant number of patients experienced a recurrence of the same adverse reaction (24%), or developed a new reaction in a different organ system (26%), upon restarting the same drug [109]. It is worth noting that the administration of a different class of checkpoint inhibitors resulted in a relatively low rate of recurring irAE. In a study conducted by Menzies et al. in 2017, it was found that among 67 patients who experienced irAE while taking ipilimumab, only 2 individuals experienced a recurrence of the same irAE after initiating anti-PD-1 inhibition [110,111]. However, a significant portion of the subjects experienced the emergence of a new irAE as a result of the treatment. However, the authors assert that the reactions were mild and that switching drug classes is a feasible and secure alternative. It is still unclear whether the same applies when moving from PD-1 to CTLA-4 inhibition. While only 70% of PD-1/PD-L1 patients have irAEs, data show that as many as 90% of patients on anti-CTLA-4 treatment do [112].

## 7. Conclusions

Until now, precision medicine has primarily focused on utilizing the molecular and genomic characteristics of tumors to prescribe targeted small molecules and biologics. However, emerging precision medicine platforms may present new possibilities for customizing treatments for individual patients or specific patient groups. As we continue to uncover the intricacies of how ICIs are responded to and resisted, we must incorporate molecular and functional technologies to create innovative precision immune-oncology platforms. The justification for using ICIs in the adjuvant setting is to eliminate micro-metastases and extend survival. However, it is crucial to not put the cart before the horse. 

## Data Availability

Not applicable.
